# Water Lung: An Interesting Case of Non-cardiogenic Pulmonary Edema in a Heart Failure Patient

**DOI:** 10.7759/cureus.48814

**Published:** 2023-11-14

**Authors:** Aqsa Iqbal, Riaz Mahmood, Manasa Jasti, Christelle Moyine, Santhi Adigopula

**Affiliations:** 1 Internal Medicine, Northeast Georgia Medical Center Gainsville, Gainesville, USA; 2 Cardiology, Northside Hospital / Northside Hospital Cardiovascular Institute, Atlanta, USA

**Keywords:** contrast dye, respiratory distress, diastolic heart failure, cardiogenic pulmonary edema, non-cardiogenic pulmonary edema

## Abstract

Contrast-induced pulmonary edema is a rare but life-threatening condition often missed in heart failure patients. We present a case of a 65-year-old female with a past medical history of coronary artery disease, diastolic heart failure, and chronic kidney disease who presented with chest pain. She received low osmolar intravenous (IV) contrast for cardiac catheterization. Within 24 hours of receiving the contrast, the patient developed respiratory distress, which was found to be secondary to pulmonary edema. Pulmonary edema was considered to be related to cardiogenic at first; however, the patient’s physical examination was normal, with no jugular venous distention (JVD). A transthoracic echocardiogram showed a central venous pressure of 3 mmHg. The patient's respiratory condition improved after receiving an IV diuretic. Chart review showed that the patient had a similar presentation in the past, which was also thought to be related to heart failure leading to recurrent exposure to contrast. Non-cardiogenic pulmonary edema should be considered in the differential diagnosis of pulmonary edema in heart failure patients receiving contrast.

## Introduction

Contrast media is used in non-invasive and invasive imaging modalities. Contrast-induced nephropathy and anaphylactic reaction are common complications of contrast media [1]. Acute severe respiratory distress secondary to non-cardiogenic pulmonary edema after infusing radiocontrast media has been rarely reported in the past.

The clinical presentation of non-cardiogenic pulmonary edema is similar to cardiogenic pulmonary edema, which leads to missing the diagnosis of non-cardiogenic pulmonary edema and significant morbidity and mortality. Our case highlights the importance of considering contrast-induced pulmonary edema in the differential diagnosis of pulmonary edema after using contrast, even if the patient has a history of heart failure.

## Case presentation

**Figure 1:**
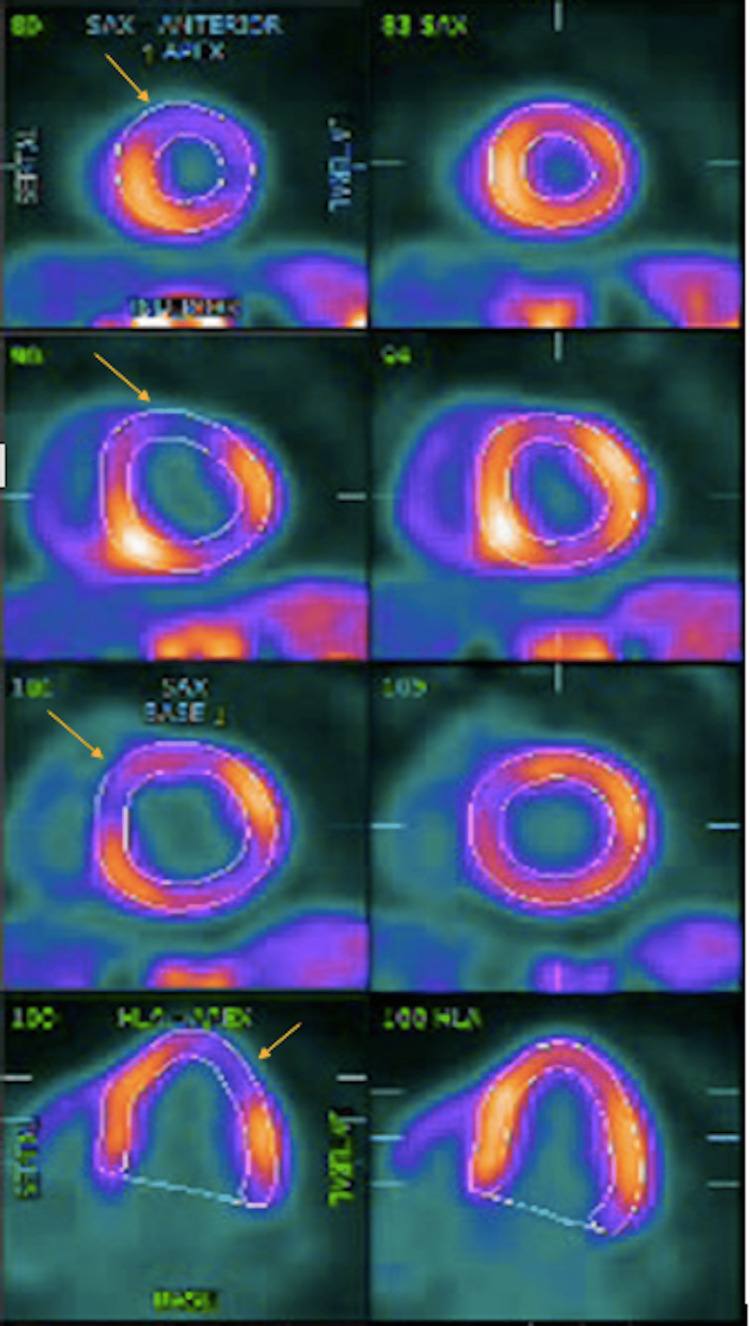
Cardiac positron tomography myocardial perfusion imaging with Lexiscan suggestive of ischemia on exercise (yellow arrows on the left) as compared to normal perfusion during rest (on the right)

A 65-year-old female presented to the emergency room with typical anginal chest pain, relieved with nitroglycerin. She denied dyspnea, lower extremity edema, orthopnea, paroxysmal nocturnal dyspnea, and weight gain. She was euvolemic on physical examination with clear breath sound, no JVD, and lower extremity edema. The electrocardiogram showed normal sinus rhythm. Transthoracic echocardiogram showed a left ventricular ejection fraction of 55-60% with grade II diastolic dysfunction and central venous pressure of 3 mmHg. Cardiac positron tomography myocardial perfusion imaging with lexiscan showed a significant reversible defect in the basal to apical, anterior wall, anteroseptal wall, and basal to apical inferolateral wall suggestive of ischemia due to occlusion of the left anterior descending artery and left circumflex artery (Figure 1).

The patient underwent a coronary angiogram with iodinated contrast, which showed a patent left internal mammary artery to the left anterior descending artery and saphenous vein graft to the obtuse marginal artery (OM1) and right coronary artery with left ventricular end-diastolic pressure of 16 mmHg. Chest pain was considered secondary to coronary vasospasm, and the patient was discharged home on medical management. The same day, the patient returned and was admitted with severe dyspnea requiring bilevel-positive airway pressure ventilation.

Past medical history

Past medical history was notable for coronary artery disease status post coronary artery bypass in 2010, diastolic heart failure, stage 3 chronic kidney disease, diabetes mellitus, hyperlipidemia, and peripheral vascular disease.

Differential diagnosis

Differential diagnoses of shortness of breath include cardiogenic pulmonary edema and non-cardiogenic pulmonary edema. Despite being euvolemic on the physical examination with normal central venous pressure, dyspnea was considered cardiogenic given her history of diastolic heart failure.

Investigations

**Figure 2:**
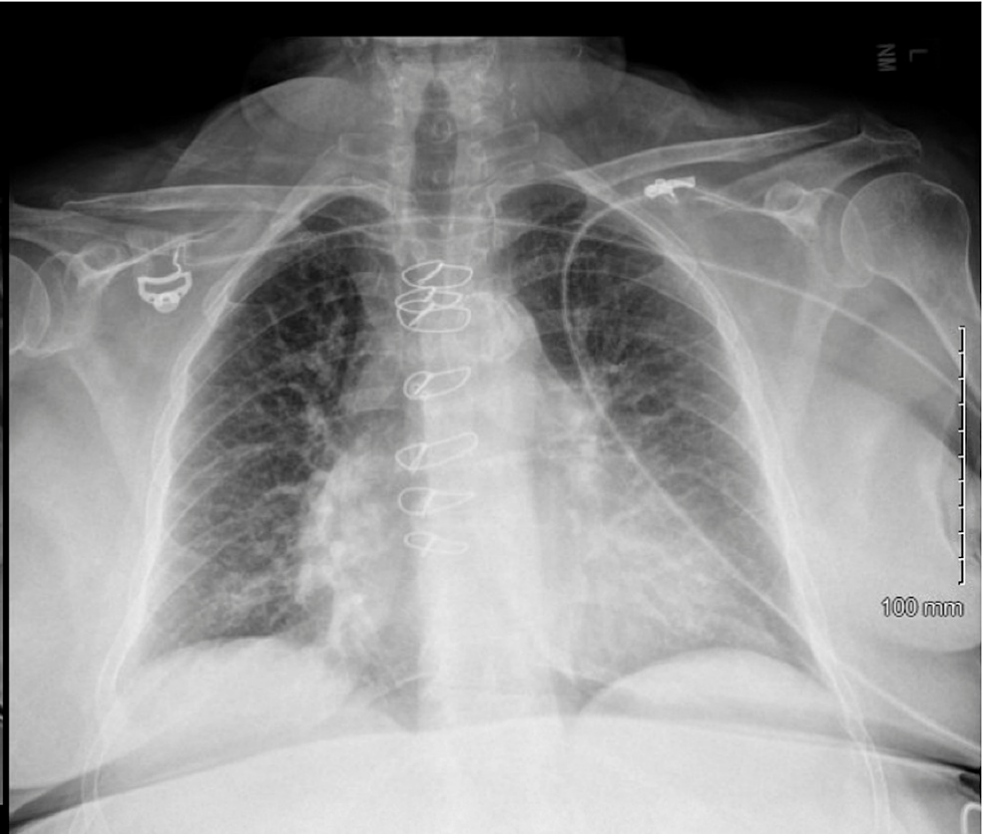
Chest X-ray before cardiac catheterization

**Figure 3:**
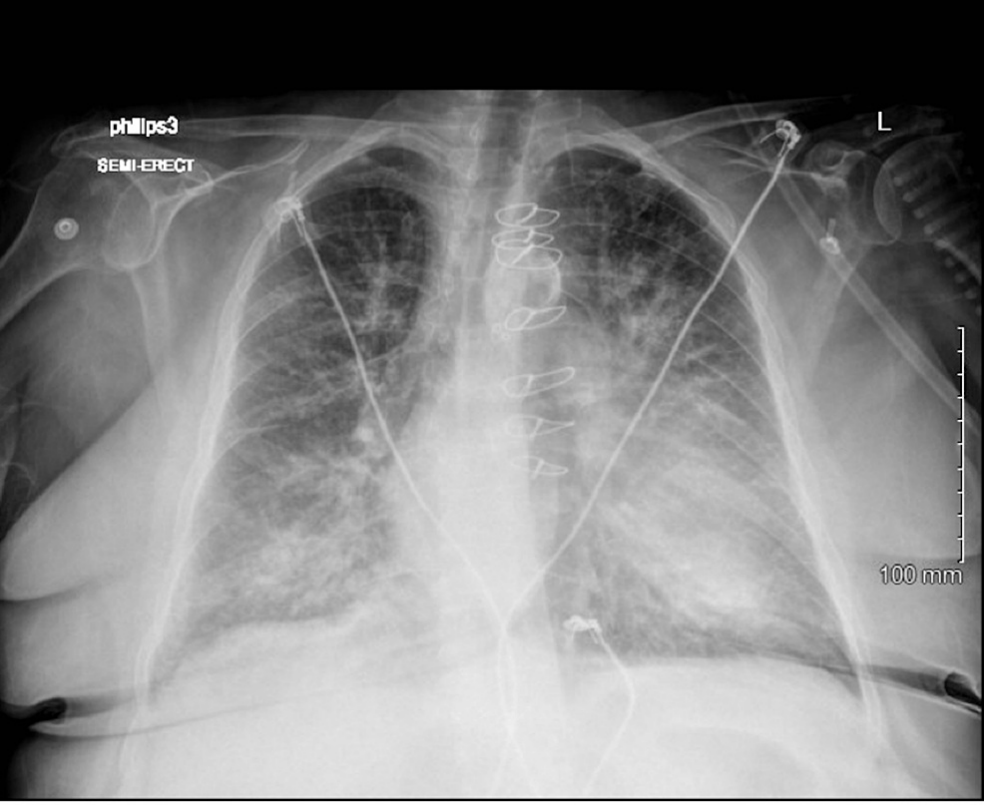
Chest X-ray after cardiac catheterization

Creatinine on the day of the coronary angiogram was 1.2 mg/dl (baseline creatinine around 1.2-1.6 mg/dl). Brain natriuretic peptide on admission was 1898 pg/ml, which increased to 9289 pg/ml on readmission. Chest X-ray before cardiac catheterization was unremarkable (Figure 2), and chest X-ray following cardiac catheterization was significant for severe pulmonary edema (Figure 3).

Management

The patient was treated with IV diuretics and showed significant improvement. Because of her diastolic heart failure history, pulmonary edema was considered to be cardiogenic. Further chart review revealed the patient had a similar presentation the year before when she was evaluated for chest pain and found to have a patent graft. She returned the same day with shortness of breath and had rales but was otherwise euvolemic on the examination. Chest X-ray showed significant pulmonary edema, which was considered to be cardiogenic. The patient’s breathing improved after one dose of IV furosemide 80 mg. Iodinated contrast was added to her allergies with a side effect of probable non-cardiogenic pulmonary edema. The patient was advised to inform healthcare providers of this allergy before undergoing imaging requiring contrast.

Follow-up

The patient had no further episodes of chest pain or hospitalizations related to non-cardiogenic pulmonary edema.

## Discussion

Contrast-induced pulmonary edema is a rare and fulminant complication of intravenous (IV) contrast [1,2]. A few case reports have noted acute respiratory failure and cardiac arrest secondary to hypoxemia due to contrast-induced pulmonary edema [3,4]. In patients with preexisting heart conditions, particularly heart failure, contrast-induced non-cardiogenic pulmonary edema can be misdiagnosed as cardiogenic edema, leading to recurrent exposure to contrast. Recurrent exposure to intravenous contrast can lead to complications, including pulmonary edema and kidney failure. Due to the high prevalence of pulmonary edema in heart failure, non-cardiogenic pulmonary edema may be considered in the differential diagnosis.

Our patient developed acute respiratory failure after receiving iodinated contrast during left heart catheterization. Although the patient appeared to be euvolemic on physical examination and echocardiogram, elevated brain natriuretic peptide (BNP) and a history of heart failure led to the diagnosis of cardiogenic pulmonary edema. The patient was treated with intravenous diuretics and discharged home. Since pulmonary edema was considered cardiogenic, the patient was exposed to intravenous contrast again without premedication with steroids and antihistamines, which could have prevented recurrent respiratory failure.

Besides premedication with steroids and antihistamines, patients with non-cardiogenic pulmonary edema must be treated with intravenous diuretics. In a case report by Nagao et al., respiratory failure after IV contrast was treated with antihistamine and steroids with no improvement until the patient received a diuretic [4].

In previous case reports of non-cardiogenic pulmonary edema following IV contrast, patients had no history of heart disease and their BNP levels were normal [5]. However, our patient had preexisting heart disease and elevated BNP levels, leading to a missed diagnosis and recurrent hospitalization.

Although our patient had a sudden significant increase in brain natriuretic peptide after receiving contrast compatible with cardiac overload, it is not apparent if the cardiac overload is secondary to contrast media or heart failure. Due to the lack of right heart catheterization in our patient, we cannot say if pulmonary edema was non-cardiogenic. However, sudden onset of respiratory distress after receiving IV contrast and euvolemia on physical examination and normal central venous pressure on an echocardiogram is more suggestive of contrast-induced pulmonary edema than heart failure.

Although previous case reports have shown that osmolality and viscosity play a significant role in non-cardiogenic pulmonary edema, our patient developed pulmonary edema despite receiving low osmolar contrast. Pincet et al. published a case report in which a 55-year-old patient developed noncardiogenic pulmonary edema after receiving low osmolar contrast for an abdominal CT scan [6].

Contrast-induced pulmonary edema is related to increased alveolar-capillary membrane permeability due to endothelial injury. The endothelial injury could be connected to direct chemotoxic, osmotic, immunologic, or allergic effects on the vascular endothelium [7,8].

Considering contrast-induced pulmonary edema as cardiogenic in heart failure can lead to misclassifying a patient as having refractory heart failure due to multiple hospitalizations. The misclassifying of patients can lead to unnecessary procedures and treatments. It can also lead to multiple hospitalizations due to recurrent exposure to contrast.

## Conclusions

Contrast-induced non-cardiogenic pulmonary edema remained undiagnosed due to premature closure, not considering all differential diagnoses and anchoring to cardiogenic pulmonary edema in the setting of heart failure. Identifying non-cardiogenic pulmonary edema in heart failure patients who recently received contrast can be challenging. Providers should avoid using contrast or premedication with steroids or antihistamines before procedures requiring contrast to prevent associated morbidity and mortality.
